# Therapeutic efficacy of liraglutide versus metformin in modulating the gut microbiota for treating type 2 diabetes mellitus complicated with nonalcoholic fatty liver disease

**DOI:** 10.3389/fmicb.2023.1088187

**Published:** 2023-01-26

**Authors:** Xing Ying, Zheng Rongjiong, Mayila Kahaer, Jiang Chunhui, Muhuyati Wulasihan

**Affiliations:** ^1^Department of Comprehensive Internal Medicine Department 4, The First Affiliated Hospital of Xinjiang Medical University, Urumqi, China; ^2^Department of Infectious Disease, The First Affiliated Hospital of Xinjiang Medical University, Urumqi, China; ^3^Department of Cardiology, The First Affiliated Hospital of Xinjiang Medical University, Urumqi, China

**Keywords:** intestinal microorganism, NAFLD, T2DM, liraglutide, metformin

## Abstract

Metformin and liraglutide are used in the treatment of type 2 diabetes mellitus (T2DM) complicated with nonalcoholic fatty liver disease (NAFLD). Although these drugs can alter the intestinal microbiome, clinical data are required to explore their mechanisms of action. Using 16S sequencing technology, we analyzed and compared the intestinal bacterial community structure and function between patients before and after treatment (12 weeks) with the two drugs (metformin or liraglutide, *n* = 15) and healthy controls (*n* = 15). Moreover, combined with 19 clinical indices, the potential therapeutic mechanisms of the two drugs were compared. The studied clinical indices included those associated with islet β-cell function (FPG, FINS, HbA1c, and HOMA-IR), inflammation (TNF-α, IL-6, and APN), lipid metabolism (TC, TG, and LDL-C), and liver function (ALT, AST, and GGT); the values of all indices changed significantly after treatment (*p* < 0.01). In addition, the effect of the two drugs on the intestinal bacterial community varied. Liraglutide treatment significantly increased the diversity and richness of the intestinal bacterial community (*p* < 0.05); it significantly increased the relative abundances of Bacteroidetes, Proteobacteria, and Bacilli, whereas metformin treatment significantly increased the relative abundance of Fusobacteria and Actinobacteria (*p* < 0.05). Metformin treatment increased the complexity and stability of the intestinal bacterial network. However, liraglutide treatment had a weaker effect on the intestinal bacterial network, and the network after treatment was similar to that in healthy controls. Correlation matrix analysis between dominant genera and clinical indicators showed that the correlation between the bacterial community and islet β-cell function was stronger after liraglutide treatment, whereas the correlation between the bacterial community and inflammation-related factors was stronger after metformin treatment. Functional prediction showed that liraglutide could significantly affect the abundance of functional genes related to T2DM and NAFLD (*p* < 0.05), but the effect of metformin was not significant. This study is the first to report the changes in the intestinal bacterial community in patients treated with metformin or liraglutide and the differences between the mechanisms of action of metformin and liraglutide. Metformin or liraglutide has a therapeutic value in T2DM complicated with NAFLD, with liraglutide having a weaker effect on the intestinal bacterial community but a better therapeutic efficacy.

## Introduction

Nonalcoholic fatty liver disease (NAFLD) is defined as the hepatic manifestation of metabolic syndrome, which is characterized by excessive ectopic lipid accumulation in the hepatocytes (Canfora et al., 2019; De Meyts and Delzenne, 2021). The prevalence of NAFLD in developed countries is approximately 25–30% and is increasing rapidly (Shabalala et al., 2020; Powell et al., 2021). Nonalcoholic fatty liver disease significantly increases the risk of type 2 diabetes mellitus (T2DM), and the prevalence of NAFLD is 70% in patients with T2DM (Bril and Cusi, 2017; Geng et al., 2021). Insulin resistance is the main driver of the interaction between NAFLD and T2DM; therefore, hypoglycemic agents have been used to treat T2DM complicated with NAFLD (Pacifico et al., 2020).

The gut microbiome plays a key role in the human body and produces specific metabolites, thereby forming complex interconnected networks with many organ systems (Leung et al., 2022). Therefore, the gut microbiome has attracted much attention as a potential target of metabolic diseases (Zhang et al., 2021). The dysfunction of the intestinal–hepatic axis, such as the structural disorder of the intestinal microbial community, the explosion of the microbial population, and the increase in intestinal permeability, can directly destroy the symbiotic relationship between the intestinal microbial community and host, leading to the host immune response dysfunction, which plays a key role in the occurrence and development of NAFLD (Saltzman et al., 2018; Yao et al., 2022).

Metformin has been the most widely prescribed prescription drug for T2DM for more than 60 years and has shown superior safety and better curative effect (Lee et al., 2021). Metformin is usually preferred to treat overweight patients with T2DM because of its weight loss benefits (Gnesin et al., 2020). It can reduce the glucose output of peripheral tissues and regulate hepatic lipid metabolism by activating AMP-activated protein kinase (Rena et al., 2013; Wu et al., 2017). Liraglutide is an analog with 97% homology to human glucagon-like peptide (GLP-1), which is another pharmaceutical approach to treat T2DM by enhancing GLP-1 function (Rabiei et al., 2021). This GLP-1 agonist binds to the receptor of the endogenous intestinal hormone GLP-1 to enhance insulin secretion and inhibit glucagon production, thereby inhibiting the development of fatty liver in patients with T2DM (Wang et al., 2017; Moreira et al., 2018).

Wang et al. (2017) were the first to compare and report the difference in the influence of commonly used hypoglycemic drugs metformin and liraglutide on intestinal flora structure. Many studies have shown that the regulation of the intestinal microbiome is a potentially important component of the mechanisms of action of metformin and liraglutide (Zhang et al., 2020; Nauck et al., 2021). However, research on the correlation between clinical treatment indexes and changes of intestinal flora structure before and after treatment with the two drugs is lacking. Therefore, on the basis of previous research, we not only compared the intestinal microbial community structure and predictive function before and after liraglutide or metformin treatment in patients with T2DM complicated with NAFLD but also performed an in-depth analysis based on the changes in the clinical parameters of patients. In addition, different from many related studies, we included healthy controls in this study. Therefore, we compared the therapeutic effects and potential mechanisms of action of the two drugs. This clinical trial aims to provide concrete evidence for the novel effects of liraglutide and metformin on the human intestinal microbiome as well as a reference for the choice of suitable clinical treatment plan for patients.

## Materials and methods

### Patient selection

From August 2018 to August 2019, 30 patients with T2DM complicated with NAFLD who were treated at the First Affiliated Hospital of Xinjiang Medical University were recruited. The research protocol was approved by the Ethics Committee of the First Affiliated Hospital of Xinjiang Medical University (Xinjiang Uygur Autonomous Region, China; No. 20181129-13). Patient selection criteria were as follows: (1) patients fulfilling the 1999 World Health Organization criteria for the diagnosis and typing of diabetes (Lipsky et al., 2020); (2) patients fulfilling the 2010 Chinese Medical Association criteria for the diagnosis of NAFLD, confirmed using B-mode ultrasound (Zhou et al., 2010); (3) those aged 18–70 years; and (4) those who provided written informed consent. Patient exclusion criteria were as follows: patients who had (1) a long-term history of alcoholism; (2) NAFLD-related diseases such as viral hepatitis and drug-induced liver disease; (3) a history of malignant hypertension, severe hyperlipidemia, or autoimmune diseases; (4) organic intestinal diseases; and (5) a history of abdominal surgery, as well as patients who were (6) pregnant or lactating; (7) intolerant to metformin; and (8) intolerant to liraglutide.

### Grouping and treatment

All patients received unified dietary guidance and exercise education. The patients were randomly divided into two groups (Met group, *n* = 15, and Lira group, *n* = 15). The Met group dosage was 1,500 mg/day, whereas the Lira group dosage was 1.8 mg/day (Lingvay et al., 2016; Tamborlane et al., 2019; Budoff et al., 2021). Both groups were given treatment for 12 weeks. The MetA and LiraA groups constituted the before-treatment groups, and the MetB and LiraB groups constituted the after-treatment groups. Fifteen healthy volunteers were recruited as healthy controls (HC group).

### Laboratory evaluation

Data on the sex, age, disease course (in patients), height, and weight of all participants were collected. Body mass index (BMI) was calculated as weight/height squared (kg/m^2^). Venous blood was collected from patients who fasted for 12 h, and the levels of fasting plasma glucose (FPG), hemoglobin A1c (HbA1c), total cholesterol (TC), triglyceride (TG), high-density lipoprotein-cholesterol (HDL-C), low-density lipoprotein-cholesterol (LDL-C), alanine aminotransferase (ALT), aspartate aminotransferase (AST), gamma-glutamyl transpeptidase (GGT), and alkaline phosphatase (ALP) were determined using a Cobas 8000 automatic biochemical analyzer (Roche, Germany). Tumor necrosis factor-α (TNF-α), interleukin 6 (IL-6), and adiponectin (APN) levels were determined using an RT-6100 enzyme immunoassay workstation (RAYTO, United states). Fasting insulin (FINS) was measured using an ARCHITECT i2000SR immunoassay analyzer (Abbott, United states; Feng et al., 2017). The homeostasis model assessment of insulin resistance (HOMA-IR) was calculated as FPG × FINS/22.5. All participants were examined using an ACUSON S2000 ultrasound diagnostic system (Siemens, Germany) on an empty stomach. Liver stiffness measurement (LSM) and controlled attenuation parameter (CAP) were measured using a FibroTouch ultrasound diagnostic instrument (Echosens, France; Shimizu et al., 2019). All measurements were performed by independent medical technicians who were blinded to the study protocol.

### Biochemical analysis

Fresh fecal samples were collected with a sterile spoon from patients in each group. The collected samples were transferred into a prelabelled tube containing 8 ml of Stool DNA Stabilizer, mixed by shaking and then immediately stored in an ultralow temperature storage (Alphavita, China) at −80°C until DNA extraction. Before DNA extraction, stool consistency was evaluated by trained laboratory technicians. Total bacterial DNA was extracted from the samples using the PowerSoil DNA Isolation Kit (MO BIO, United states). Using NanoDrop^™^ One (Thermo Fisher, United states) and BioPhotometer D30 (Eppendorf, Germany), the quality and quantity of the extracted DNA were determined. The V3–V4 region of the bacterial 16S rRNA gene was amplified using a primer pair (338F, 806R). The sequencing and bioinformatics services of all the samples were completed on the Illumina Hiseq 2,500 platform of BMK Cloud (www.biocloud.net, Biomarker Technologies Co. Ltd., Beijing, China; Chen et al., 2021). Sequence read archive (SRA) records will be accessible with the following link after the indicated release date: https://www.ncbi.nlm.nih.gov/sra/PRJNA896892.

Flash (v. 1.2.11) and Trimmomatic (v. 0.33) software were used to obtain high-quality reads. UCHIME (v. 8.1) software was used to identify and remove the chimeric sequences and obtain the final data. USEARCH (v. 10.0) software was used to cluster the reads (at a similarity level of 97%) to obtain the operational taxonomic units (OTUs), and the OTUs were taxonomically annotated based on the 16S bacterial taxonomy database (Silva, release 132). The RDP classifier was used to assign taxonomic groups (a minimal confidence estimate of 80%). The microbial community diversity was analyzed using mothur (v. 1.30). The KEGG database was used to predict the function of the microbial community (Chen et al., 2021).

### Statistical analyses

All data analysis and drawing were performed using R (v. 4.0.5) and the ggplot2 package (v. 3.3.5). Pairing and multiple comparisons were performed with the agricolae package (v. 1.3-5) and ggpubr package (v. 0.4.0). The nonmetric multidimensional scaling (NMDS) analysis was performed using the vegan package (v. 2.5-7). Stacked column diagram was prepared using the ggalluvial package (v. 0.12.3). The linear discriminant analysis effect size (LEfSe) was completed using the microeco package (v. 0.7.1). The network analysis was performed using the phyloseq package (v. 1.39.1), ggClusterNet package (v. 0.1.0), igraph package (v. 1.2.11), and Gephi (v. 0.9.7). Correlation matrix analysis was performed using the linkET package (v. 0.0.2.9; Chen et al., 2021).

## Results

### Effects of metformin on liraglutide on clinical indices

After treatment with the two drugs, the values of HDL-C, ALP, and LSM did not change significantly (*p* > 0.05), but other indices changed significantly (*p* < 0.01; Table 1). Unlike in the Met group, the values of BMI, HOMA-IR, TC, and LDL-C in the Lira group changed significantly (*p* < 0.001). In addition, the MetB, LiraB, and HC groups were compared. The weight, BMI, FPG, HbA1c, TG, ALT, AST, GGT, ALP, CAP, and LSM of patients in the Met and Lira groups all decreased after treatment, but these values were still significantly higher than those in the HC group, and TG, ALT, and AST levels in the Lira group were significantly lower than those in the MetB group (*p* < 0.05). Meanwhile, HOMA-IR, IL-6, TC, and LDL-C levels did not differ significantly between the HC and LiraB groups, but the highest values were observed in the MetB group. In addition, FINS and TNF-α levels in the Met and Lira groups decreased significantly (*p* < 0.001), which showed no significant difference with those in the HC group.

**Table 1:** Changes of clinical indices before and after msetformin or liraglutide treatment and comparison with healthy controls.

	MetA	MetB	*p*-Value 1	LiraA	LiraB	*p*-Value 2	HC
Number (M/F)	15 (7, 8)	–		15 (8, 7)	–		15 (8, 7)
Disease course (year)	4.93 ± 0.35	–		5.33 ± 0.42	–		–
Weight (kg)	86.47 ± 1.82	84.58 ± 0.52 a		87.8 ± 2.01	81.34 ± 1.91 a		66.86 ± 1.41 b
BMI (kg/m^2^)	31.67 ± 0.27	29.26 ± 0.60 a	**	31.42 ± 0.28	28.53 ± 0.44 a	***	22.36 ± 0.41 b
FPG (mmol/L)	9.22 ± 0.42	6.97 ± 0.05 a	***	9.50 ± 0.43	6.79 ± 0.41 a	***	5.52 ± 0.13 b
FINS (mU/L)	59.06 ± 5.19	12.36 ± 0.34 a	***	61.65 ± 6.69	12.88 ± 1.52 a	***	10.25 ± 0.57 a
HbA1c (%)	9.75 ± 0.17	7.56 ± 0.07 a	***	9.78 ± 0.19	7.27 ± 0.15 a	***	5.68 ± 0.11 b
HOMA-IR	9.03 ± 1.02	5.14 ± 0.06 a	**	8.38 ± 0.89	3.65 ± 0.30 b	***	3.76 ± 0.01 b
TNF-α (ng/ml)	61.72 ± 1.81	46.29 ± 0.45 a	***	62.46 ± 1.80	46.86 ± 1.72 a	***	44.16 ± 0.27 a
IL-6 (pg/ml)	51.63 ± 1.60	38.84 ± 0.28 a	***	50.54 ± 1.80	36.93 ± 0.97 b	***	35.66 ± 0.46 b
APN (mg/L)	6.84 ± 0.12	8.05 ± 0.04 b	***	6.92 ± 0.17	8.10 ± 0.18 b	***	11.8 ± 0.59 a
TC (mmol/L)	5.34 ± 0.15	4.84 ± 0.05 a	**	5.27 ± 0.14	4.47 ± 0.13 b	***	4.45 ± 0.04 b
TG (mmol/L)	4.09 ± 0.28	3.21 ± 0.05 a	**	3.99 ± 0.34	2.49 ± 0.23 b	**	1.51 ± 0.03 c
HDL-C (mmol/L)	0.95 ± 0.01	0.96 ± 0.00 b		0.96 ± 0.02	0.97 ± 0.02 b		1.25 ± 0.02 a
LDL-C (mmol/L)	4.13 ± 0.10	3.77 ± 0.03 a	**	4.11 ± 0.11	2.81 ± 0.08 b	***	2.82 ± 0.05 b
ALT (U/L)	66.65 ± 1.77	51.81 ± 0.85 a	***	63.81 ± 2.16	47.73 ± 1.10 b	***	24.76 ± 0.08 c
AST (U/L)	85.64 ± 1.33	61.72 ± 0.73 a	***	83.33 ± 1.83	57.82 ± 0.87 b	***	24.2 ± 0.06 c
GGT (U/L)	98.52 ± 1.51	77.04 ± 0.78 a	***	98.99 ± 1.52	75.79 ± 0.74 a	***	39.18 ± 1.74 b
ALP (U/L)	72.87 ± 3.87	69.85 ± 1.03 a		71.96 ± 3.89	67.94 ± 3.82 a		36.4 ± 0.63 b
CAP (db/m)	280.38 ± 3.24	246.38 ± 2.81 a	***	281.21 ± 3.43	245.61 ± 3.22 a	***	217.51 ± 4.37 b
LSM (kPa)	9.97 ± 0.40	9.48 ± 0.15 a		10.37 ± 0.33	9.58 ± 0.40 a		7.06 ± 0.04 b

Mean ± standard error (*n* = 15). Different letters indicate significant differences between the MetB, LiraB, and HC groups at the level of 0.05. “**” and “***” indicate significant differences at the levels of 0.01 and 0.001 before and after a single drug treatment.

### Effects of metformin or liraglutide on the diversity of the intestinal bacterial community

**Figure 1:**
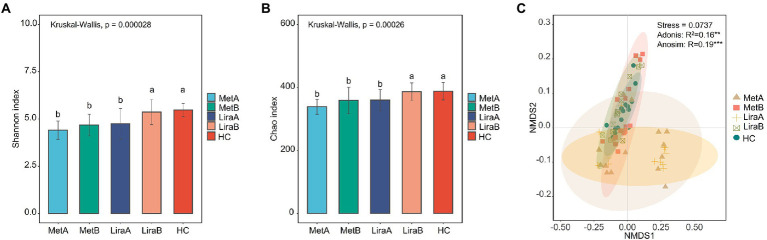
**(A)** Difference in the Shannon diversity index of the intestinal bacterial community between healthy controls and patients before and after metformin or liraglutide treatment. **(B)** Difference in the Chao richness index of the intestinal bacterial community between healthy controls and patients before and after metformin or liraglutide treatment. **(C)** β-diversity of the intestinal bacterial community in healthy controls and patients before and after metformin or liraglutide treatment. Different letters indicate significant differences between groups. *p* value < 0.05 indicates that the differences are significant. “**” and “***” indicate significant differences at the levels of 0.01 and 0.001.

Before treatment, the Shannon diversity index and Chao1 richness index of the intestinal bacterial community in the MetA and LiraA groups were significantly lower than those in the HC group. After treatment, compared with the MetA group, the α-diversity index of the intestinal bacterial community in the MetB group increased but not significantly, whereas that in the LiraB group was significantly higher than that in the LiraA group and close to that in the HC group (Figures 1A,B). The NMDS analysis showed that the stress function value was 0.0737 (< 0.1), and the sorting model was reasonable. Analysis of similarities (ANOSIM; *p* < 0.001) and Adonis analysis (*p* < 0.01) results were significant. The samples in each group had good aggregation, and there were obvious differences among the groups. The sample spacing between the MetB, LiraB, and HC groups was small, whereas that between the MetA, LiraA, and HC groups was large (Figure 1C).

### Effects of metformin or liraglutide on the intestinal bacterial community structure

**Figure 2:**
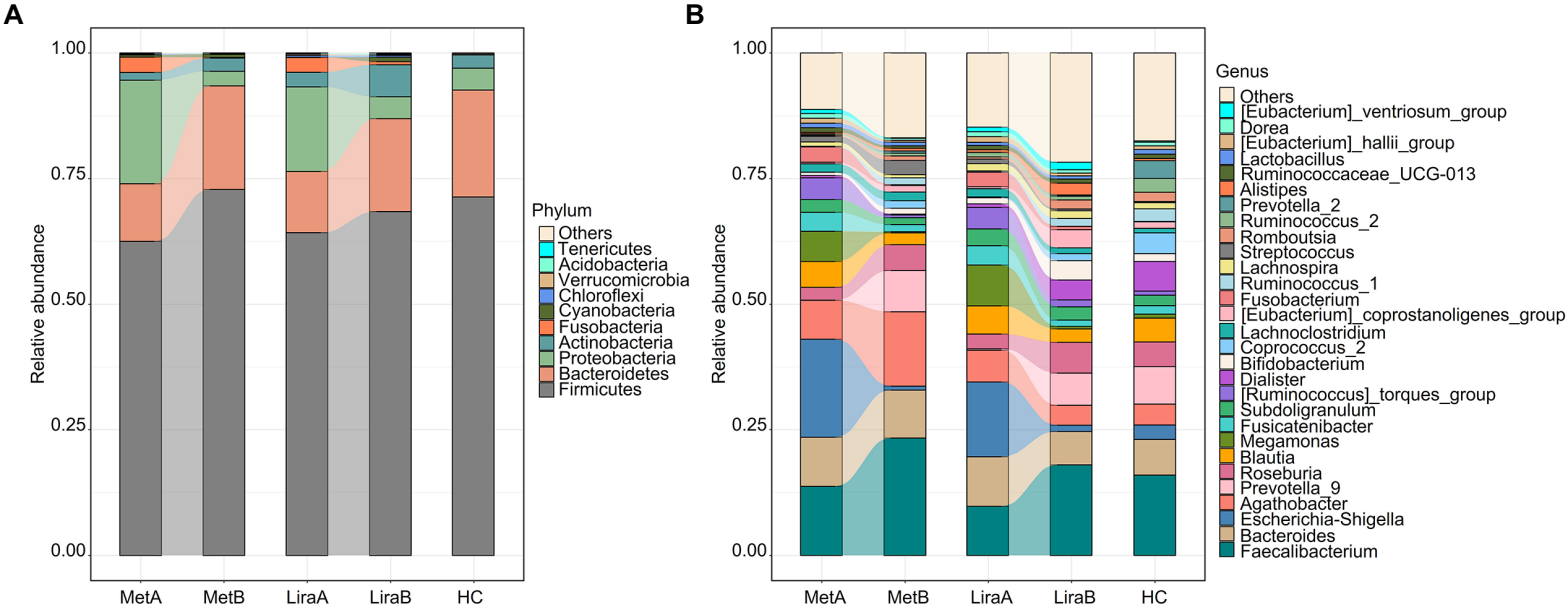
**(A)** Differences in the intestinal bacterial community structure (phylum level) between healthy controls and patients before and after metformin or liraglutide treatment. **(B)** Differences in the intestinal bacterial community structure at the genus level between healthy controls and patients before and after metformin or liraglutide treatment.

At the phylum level, Firmicutes (62.52–72.84%), Bacteroidetes (11.44–21.29%), and Proteobacteria (2.92–20.62%) were predominant in the intestinal bacterial community (Figure 2A). At the phylum level, Firmicutes (62.52–72.84%), Bacteroidetes (11.44–21.29%), and Proteobacteria (2.92–20.62%) were predominant in the intestinal bacterial community (Figure 2A). The relative abundances of Firmicutes, Bacteroidetes, and Actinobacteria in the MetA group were 62.52, 11.44, and 1.55%, respectively, which increased to 72.84, 20.61, and 2.62%, respectively, in the MetB group. The relative abundances of Proteobacteria and Fusobacteria in the MetA group were 20.62 and 3.05%, respectively, which decreased to 2.92 and 0.14%, respectively, in the MetB group. The relative abundances of Firmicutes, Bacteroidetes, and Actinobacteria in the LiraA group were 64.24, 12.15, and 2.91%, respectively, which increased to 68.46, 18.46, and 6.34%, respectively, in the LiraB group. The relative abundances of Proteobacteria and Fusobacteria in the LiraA group were 16.86 and 2.95%, respectively, which decreased to 4.38 and 0.65%, respectively, in the LiraB group. The relative abundance of the main bacteria showed the same trend after treatment with the two drugs. The relative abundances of Firmicutes, Bacteroidetes, Proteobacteria, Actinobacteria, and Fusobacteria in the HC group were 71.35, 21.29, 4.35, 2.63, and 0.04%, respectively, which were similar to the bacterial community structure of the MetB and LiraB groups.

Changes in the community structure at the genus level are presented in Figure 2B. *Faecalibacterium* (9.78–23.36%), *Bacillus* (6.65–9.84%), *Escherichia*–*Shigella* (0.78–19.51%), and *Agaricus* (3.97–14.80%) were the predominant bacteria in the intestinal bacterial community. The relative abundances of *Faecalibacterium*, *Prevotella*_9, and *Roseburia* increased to 23.36, 8.23, and 5.13%, respectively, in the MetB group and 18.02, 6.39, and 6.16%, respectively, in the LiraB group. These values were higher than the corresponding values in the HC group (16.00, 7.43 and 4.92%). The relative abundances of *Bacillus*, *Escherichia*–*Shigella*, *Blautia*, and *Megamonas* decreased were 9.54, 0.78, 2.34, and 0.23%, respectively, in the MetB group and 6.65, 1.24, 2.63, and 0.54%, respectively, in the LiraB group. The relative abundance of *Agathobacter* in the dominant genera was significantly different after treatment with the two drugs; the values changed from 7.73% (MetA) to 14.80% (MetB) and from 6.32% (LiraA) to 3.97% (LiraB). The relative abundance of *Agathobacter* in the HC group was 4.18%. Simultaneously, more differences in the changes of some genera with low relative abundances were observed. The relative abundance of *Dialister* in the HC group was 5.88%, increasing from 0.70% (LiraA) to 3.94% (LiraB) and decreasing from 0.45% (MetA) to 0.18% (MetB). The relative abundance of *Streptococcus* (2.83%) in the MetB group was higher, and the relative abundances of *Alistipes* (2.33%), the [*Eubacterium*]_*coprostanoligenes*_group (3.60%), *romboutsia* (1.81%), and others (21.76%) in the LiraB group were higher.

**Figure 3:**
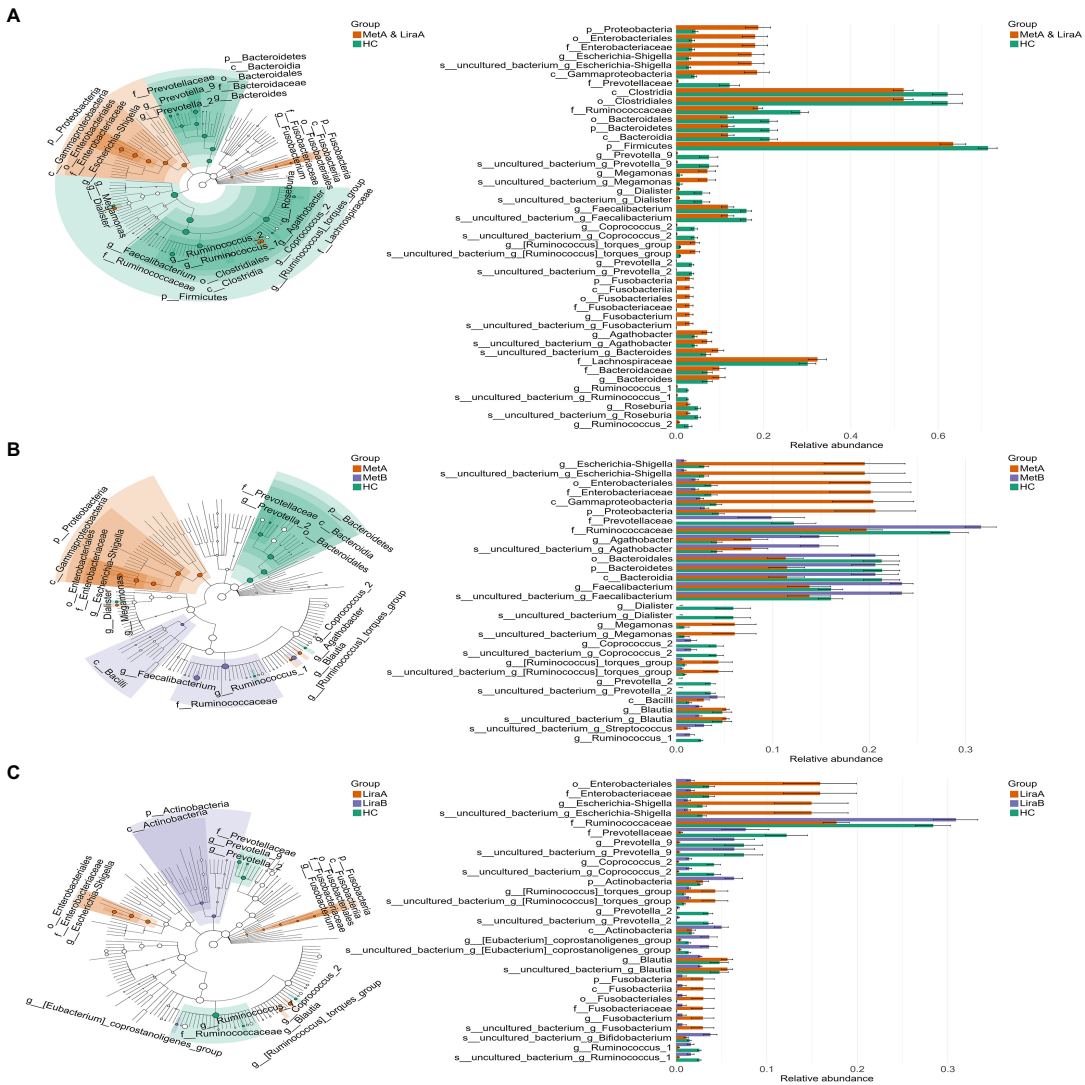
**(A)** Linear discriminant analysis effect size (LEfSe) of the intestinal bacterial community structure in healthy controls and patients before treatment (MetA & LiraA groups). **(B)** LEfSe of the intestinal bacterial community structure of patients before and after metformin treatment and healthy controls. **(C)** LEfSe of the intestinal bacterial community structure of patients before and after liraglutide treatment and healthy controls.

We observed differences in the bacterial groups at the phylum to species levels by LEfSe analysis (LDA = 4.0; Figure 3). Comparing the pre-treatment (MetA and LiraA) and HC groups, 4 phyla (Proteobacteria, Bacteroidetes, Firmicutes, and Fusobacteria), 4 classes, 4 orders, 6 families, 14 genera (including *Escherichia*–*Shigella*, *Prevotella*_9, and *Megamonas*), and 13 species were present. Comparing the HC group with the MetA and MetB groups, 4 phyla (Proteobacteria, Bacteroidetes, Firmicutes, and Fusobacteria), 4 classes, 4 orders, 6 families, 14 genera (including *Escherichia*–*Shigella*, *Agathobacter*, and *Faecalibacterium*), and 13 species were present. Significant differences were observed among the groups. Comparing the HC group with the LiraA and LiraB groups, 2 phyla (Actinobacteria and Fusobacteria), 2 classes, 2 orders, 4 families, and 9 genera (including *Escherichia*–*Shigella*, *Prevotella*_9, and *Coprococcus*_2) were present, and significant differences were observed among ten species (*p* < 0.05). Differences were observed in the effects of the two drugs on intestinal bacterial groups at various levels, especially at genus and species levels.

### Effects of metformin or liraglutide on the intestinal bacterial network

**Figure 4:**
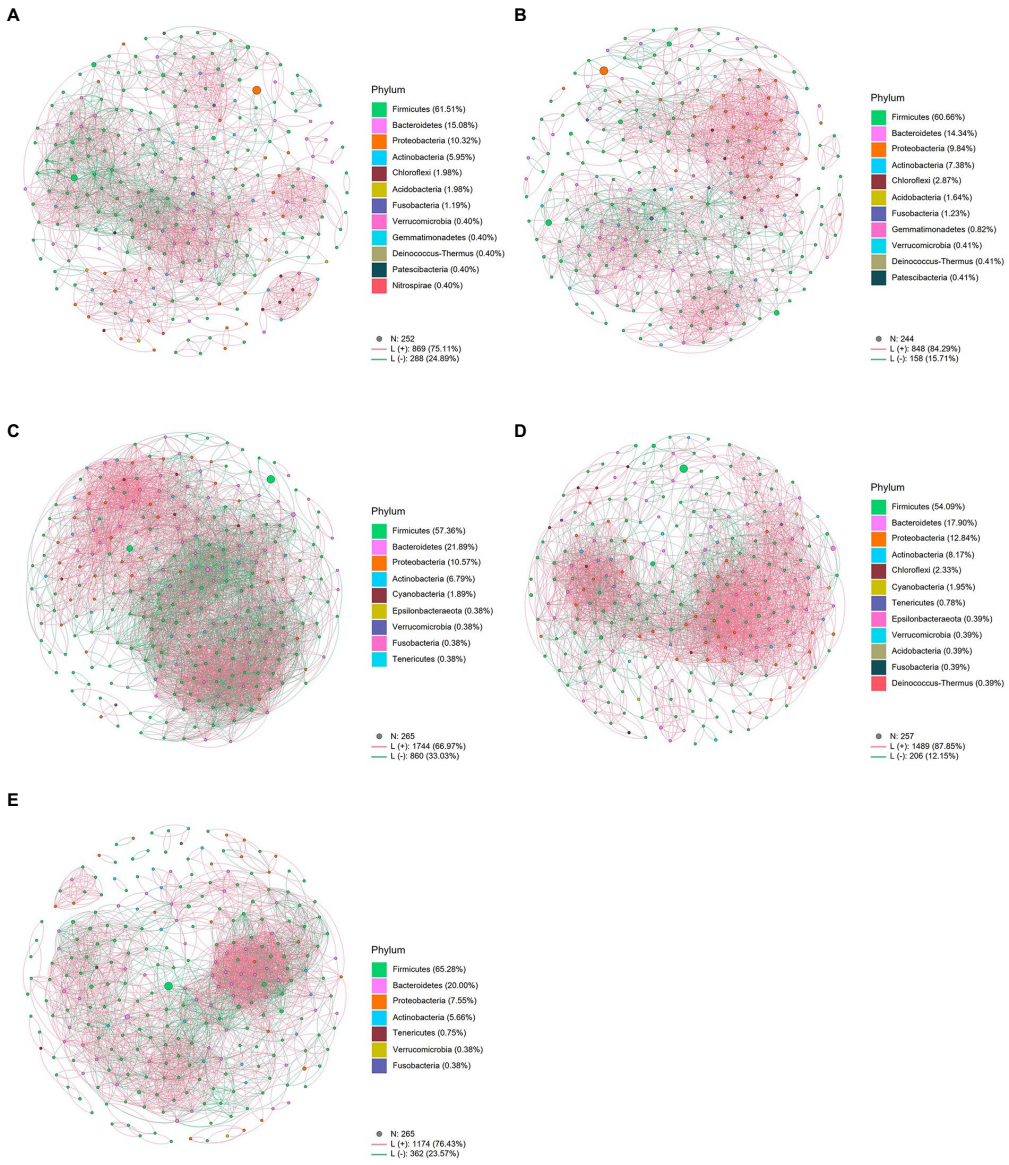
**(A)** Network analysis of the intestinal bacterial community in patients before metformin treatment. **(B)** Network analysis of the intestinal bacterial community in patients before liraglutide treatment. **(C)** Network analysis of the intestinal bacterial community in patients after metformin treatment. **(D)** Network analysis of the intestinal bacterial community in patients after liraglutide treatment. **(E)** Network Analysis of the intestinal bacterial community in healthy controls.

**Figure 5:**
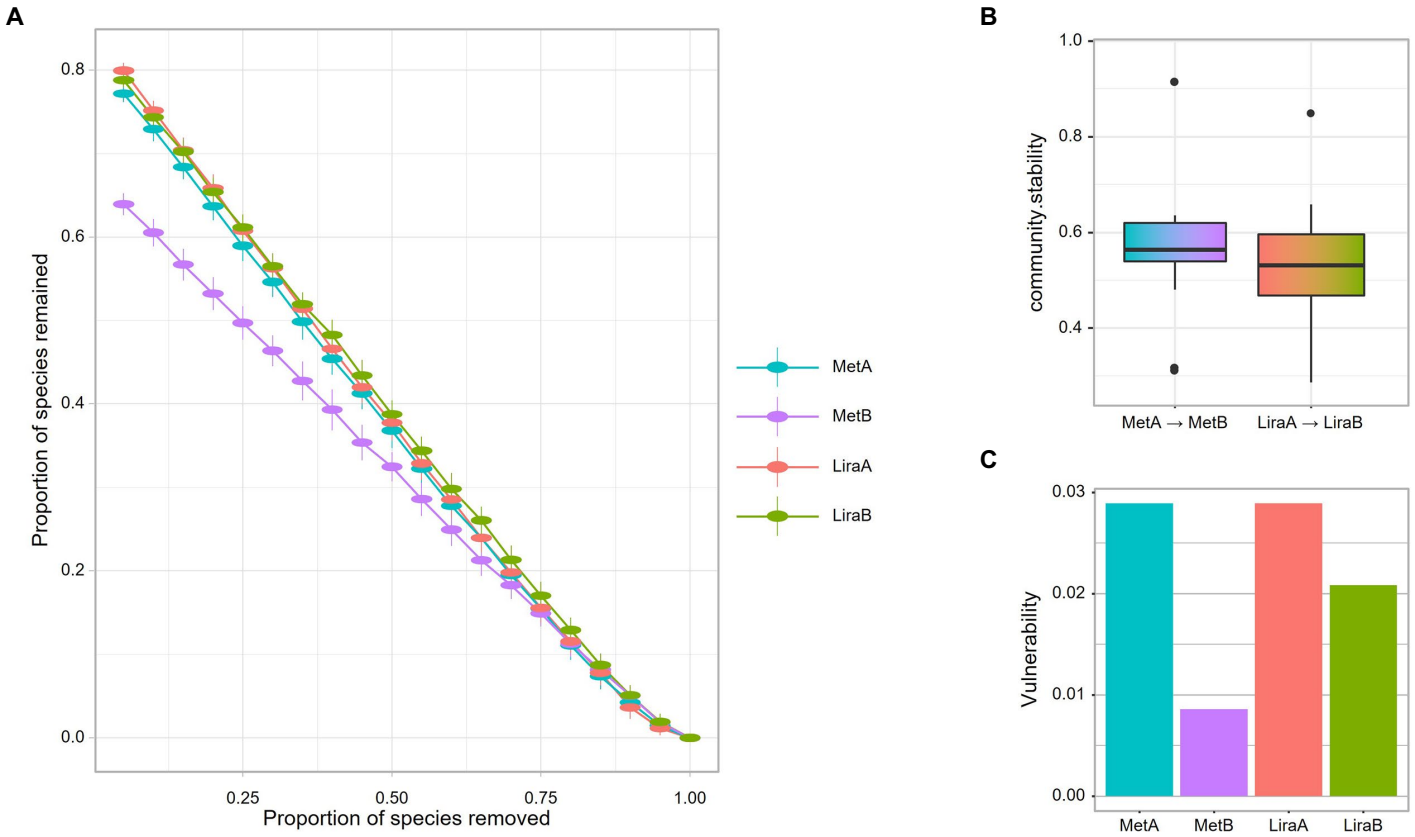
**(A)** Network robustness after exclusion of a proportion of species before and after metformin or liraglutide treatment. **(B)** Network community stability before and after metformin or liraglutide treatment. **(C)** Network vulnerability before and after metformin or liraglutide treatment.

For the intestinal bacterial community network analysis, we screened 300 OTUs with the highest abundance (Figure 4). The network of the MetB group was denser and more complex than that of the MetA group (Figures 4A,C). Interestingly, compared with the LiraA group, the bacterial network of the LiraB group was similar to that of the HC group (Figures 4B,D,E). By comparing the parameters of the bacterial community networks of the MetB and LiraB groups, we found that positive edges, negative edges, and vertices, were increased in both groups, and the edges of the MetB group were 2.25 times those of the MetA group (Table 2). The treatment with the two drugs increased the connectivity and average degree of the network but decreased the diameter and average path length. Furthermore, the clusters and the mean clustering coefficient of the MetB group network were reduced compared with those of the MetA group. However, the LiraB group network clusters were not decreased, and the mean clustering coefficient increased after drug treatment. Moreover, the number of keystone nodes in the LiraB group increased from 17 (MetA group) to 31, whereas that in the MetB group only increased from 9 (MetA group) to 11. These results showed that both treatments increased the complexity of the network, and the bacterial network in the Met group was aggregated and complicated. Though the network structure of the Lira group changed slightly, its keystone nodes were increased. In addition, we analyzed the stability of the network. The analysis of robust network composition stability after removing any proportion of species showed that metformin affected the stability of the intestinal bacterial community composition more strongly than liraglutide did (Figures 5A,B). The network vulnerability results showed that the vulnerability of the MetB group network was reduced (Figure 5C), verifying that the MetB group network was aggregated.

**Table 2:** Topological characteristics of intestinal bacterial community network before and after metformin or liraglutide treatment and in healthy controls.

Network characteristics	MetA	MetB	LiraA	LiraB	HC
num.edges (L)	1,157	2,604	1,006	1,695	1,536
num.pos.edges	869	1744	848	1,489	1,174
num. Neg. edges	288	860	158	206	362
num.nodes (n)	252	265	244	257	265
Connectance (edge_density)	0.037	0.074	0.034	0.052	0.044
average.degree (Average K)	9.183	19.653	8.246	13.191	11.592
average.path.length	4.158	2.879	4.496	3.636	3.795
diameter	9.251	7.338	12.383	8.352	9.904
no.clusters	15	10	15	15	23
mean.clustering.coefficient (Average. CC)	0.527	0.488	0.453	0.502	0.503
the.number.of.keystone.nodes	9	11	17	31	11

### Correlation analysis of clinical indicators and intestinal bacterial community after metformin or liraglutide treatment

**Figure 6:**
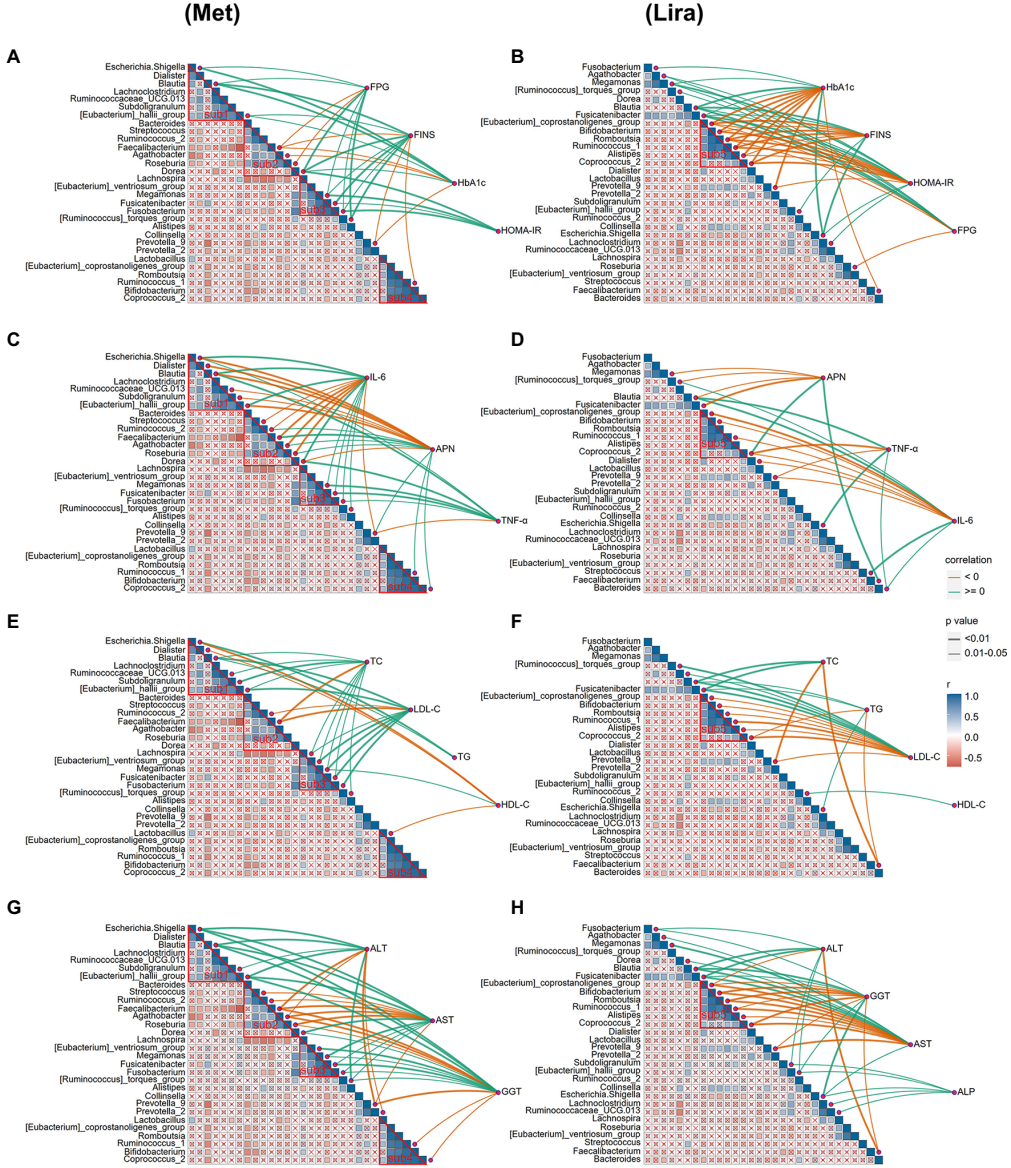
**(A)** Correlation analysis of islet β cell indices and intestinal bacterial community after metformin treatment. **(B)** Correlation analysis of islet β cell indices and intestinal bacterial community after liraglutide treatment. **(C)** Correlation analysis of inflammation-related factors and intestinal bacterial community after metformin treatment. **(D)** Correlation analysis of inflammation-related factors and intestinal bacterial community after liraglutide treatment. **(E)** Correlation analysis of lipid metabolism indices and intestinal bacterial community after metformin treatment. **(F)** Correlation analysis of lipid metabolism indices and intestinal bacterial community after liraglutide treatment. **(G)** Correlation analysis of liver function indices and intestinal bacterial community after metformin treatment. **(H)** Correlation analysis of liver function indices and intestinal bacterial community after liraglutide treatment.

The results of the autocorrelation analysis of changes in the physical and chemical properties after drug treatment showed that the four sub-communities (sub1, 2, 3, and 4) of the intestinal bacterial community in the Met group were significantly autocorrelated (Figure 6A). However, only one subcommunity (sub5) of the intestinal bacterial community in the Lira group showed significant autocorrelation (*p* < 0.05), which was similar to the bacterial group contained in sub4 of the Met group (Figure 6B).

We further analyzed the correlation between changes in the physical and chemical properties and the intestinal bacterial community after drug treatment. The correlation analysis showed that the correlation between the β-cell function index and intestinal bacterial community in the Lira group (44) was significantly stronger than that in the Met group (29; Figures 6A,B). The number of bacterial groups significantly related to FPG, FINS, HbA1c, and HOMA-IR was 8, 10, 7, and 4, respectively, in the Met group, and 8, 10, 13, and 13, respectively, in the Lira group (p < 0.05). Interestingly, in the Met group, only 4 of the 10 bacterial groups that were significantly related to FINS (*Blautia*, *Fusicatenibacter*, *Ruminococcus*_1, and [*Eubacterium*]_hallii_group) also appeared in the Lira group. In the Lira group, only 4 of the 13 bacterial groups that were significantly related to HbA1c (*Blautia*, *Escherichia*–*Shigella*, *Faecalibacterium*, and *Prevotella*_9) also appeared in the Met group.

The correlation analysis between inflammatory factors and the intestinal bacterial community showed that the correlation of the Met group (35 lines) was significantly stronger than that of the Lira group (21 lines; Figures 6C,D). Mainly, 14 and 6 bacterial groups in the Met and Lira groups, respectively, were significantly related to APN, IL-6, and TNF-α. In the Met group, only 3 of the 6 bacterial groups that were significantly related to APN (*Blautia*, *Coprocccus* _ 2, and *Faecalibacterium*) also appeared in the Lira group. In the Met group, only 4 of the 10 bacterial groups that were significantly related to IL-6 (*Blautia*, *Prevotella*_9, *Streptococcus*, [*Ruminococcus*]_torques_group) also appeared in the Lira group. In contrast, a significant positive correlation was observed between *Streptococcus* and IL-6 in the Met group (*r* ≥ 0, *p* < 0.01), whereas a significant negative correlation between them was observed in the Lira group (*r* < 0, *p* < 0.05).

The correlation between the lipid metabolism index and intestinal bacterial community in the Met group (25 lines) was significantly stronger than that of the Lira group (21 lines; Figures 6E,F). The number of bacterial groups significantly related to HDL-C, LDL-C, TC, and TG was 4, 9, 10, and 2, respectively, in the Met group and 1, 11, 4, and 5, respectively, in the Lira group. Comparatively, changes in the intestinal bacterial community in the Met group were more closely related to changes in HDL-C and TC than those in the Lira group. However, change in the intestinal bacterial community in the Lira group was not closely related to HDL-C but was more closely related to TG than that in the Met group.

Changes in liver function indices in the Met (38 lines) and Lira groups (37 lines) were closely related to changes in the intestinal bacterial community (Figures 6G,H). The number of bacterial groups significantly related to ALT, AST, GGT, and ALP was 9, 14, 15, and 0, respectively, in the Met group and 7, 13, 12, and 5, respectively, in the Lira group. The bacterial groups significantly related to ALT and AST differed between the Met and Lira groups. However, the bacterial groups significantly related to GGT were the same in the Met and Lira groups. Interestingly, 5 bacterial groups (*r* ≥ 0, *p* < 0.05) were found in the Lira group, which were not present in the Met group. To summarize, based on the correlation analysis between the physical and chemical properties and intestinal bacteria after drug treatment, obvious differences were observed between the two drug groups.

### Effects of metformin or liraglutide on intestinal bacterial community functions in patients

**Figure 7:**
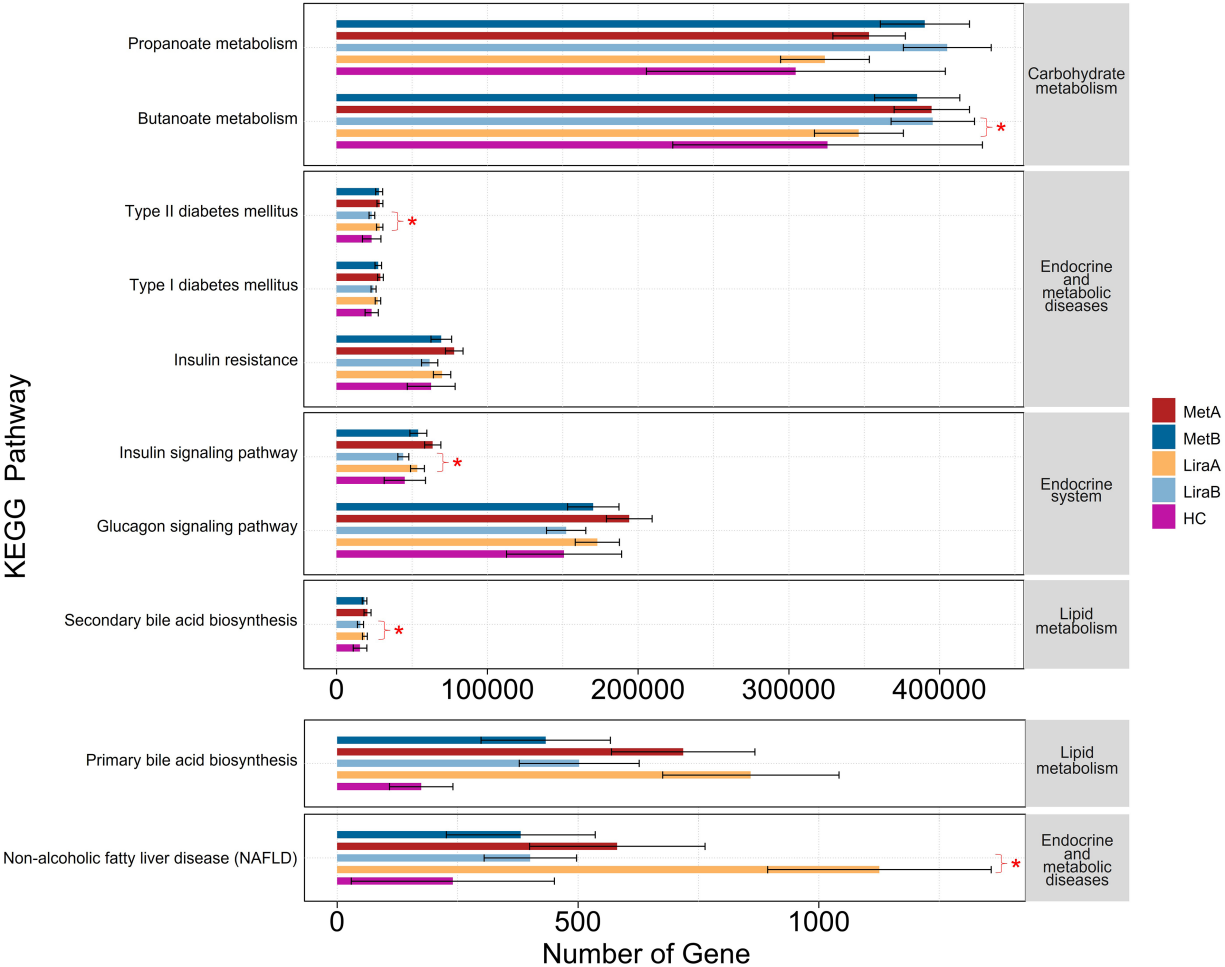
Differences in intestinal bacterial community function between healthy controls and patients before and after metformin or liraglutide treatment. “*” indicates significant differences at 0.05 level between the groups.

The results of functional prediction showed that after treatment with the two drugs, the abundances of various functional genes in the intestinal bacterial community differed between the groups (Figure 7). Regarding carbohydrate metabolism, the expression abundance of propanoate metabolism-related genes in the MetA and LiraA groups was higher than that in the HC group, which was further increased after drug treatment. The expression of butanoate metabolism-related genes in the LiraB group was significantly higher than that in the LiraA group (*p* < 0.05), whereas the expression of these genes in the MetB group was lower than that in the MetA group. Regarding endocrine and metabolic diseases, the expression of related functional genes in the HC group was low. The expression of functional genes of type 2 diabetes mellitus (T2DM) and insulin resistance decreased in the MetB and LiraB groups, whereas the expression of T2DM-related functional genes in the LiraB group was significantly lower than that in the LiraA group (*p* < 0.05). A similar trend of gene expression changes was observed in the genes associated with the endocrine system (insulin signaling and glucagon signaling pathways), lipid metabolism (primary and secondary bile acid biosynthesis), endocrine and metabolic diseases, and nonalcoholic fatty liver disease (NAFLD). Furthermore, the expressions of genes associated with the insulin signaling pathway, secondary bile acid biosynthesis, and NAFLD in the LiraB group were significantly lower than those in the LiraA group (*p* < 0.05).

## Discussion

Nonalcoholic fatty liver disease pathogenesis and prevention strategies have become the research focus globally. Many studies have confirmed the therapeutic efficacy of hypoglycemic agents in T2DM complicated with NAFLD (Ferguson and Finck, 2021). Based on islet β-cell function, Tian et al. (2018) found that the FPG, HbA1c, and HOMA-IR levels in patients with T2DM complicated with NAFLD decreased after treatment with liraglutide and metformin for 12 weeks, and liraglutide had a better therapeutic effect than metformin. Liraglutide can promote insulin secretion, inhibit glucagon release, and significantly improve patient metabolism. In this study, after treatment with metformin and liraglutide, the FPG, FINS, HbA1c, and HOMA-IR levels in patients were significantly decreased (*p* < 0.01), and the therapeutic effect of liraglutide in terms of FINS and HOMA-IR levels was significantly better than that of metformin, consistent with previous research results (Tian et al., 2018). Related studies have reported that metformin can be used to reduce the weight and fat distribution in patients; however, some studies have shown that the effect is not significant (Legro et al., 2013). Liraglutide can reduce liver fat and TG contents and thus help in treating obesity (Kuchay et al., 2020). Studies based on animal models have also confirmed that liraglutide can reduce liver lipid content and treat NAFLD (Moreira et al., 2018). According to the results of the present study, both drugs can reduce the body weight and BMI of patients, with the effect of liraglutide being superior to that of metformin. Regarding lipid metabolism, both drugs significantly reduced TC, TG, and LDL-C levels in patients (*p* < 0.01), and no significant difference in the TC and LDL-C levels were observed between patients treated with liraglutide and HC (*p* > 0.05). These results show that liraglutide is superior to metformin in improving lipid metabolism in patients. We, thus, infer that the two drugs have no significant effect on the HDL-C levels (*p* > 0.05), consistent with the results of Feng et al. (2017). However, Seif El-Din et al. (2021) showed that metformin significantly reduced HDL-C levels in mice, and the discrepancy in the result might be attributed to different subjects considered in both studies. Insulin resistance plays an vital role in NAFLD pathogenesis, and various inflammatory factors such as IL-6 and TNF-α can promote insulin resistance, leading to NAFLD occurrence and development (Tanase et al., 2020). APN activation induces the AMP-activated protein kinase pathway, reduces proinflammatory cytokines and gluconeogenesis, prevents insulin resistance, and inhibits liver inflammation (Ahmad et al., 2019). In this study, based on inflammatory factors, liraglutide was more beneficial to the liver function of the patients as it significantly decreased the IL-6 level in patients. To determine the liver functions, Feng et al. (2017) reported that after the treatment of patients with metformin or liraglutide, the level of liver injury markers, namely ALT and AST, decreased by 23.06/44.86% and 33.59/44.25%, respectively. In this study, after treatment with metformin and liraglutide, the levels of ALT, AST, and GGT significantly decreased by 22.27%/25.20, 27.93%/30.61, and 21.80%/23.44%, respectively, (*p* < 0.01). The results of this study are consistent with those of a previous study (Feng et al., 2017), and the difference in the amplitude of change can be ascribed to the different dosages of drugs. Moreover, we detected and compared the CAP and LSM of the patients. After treatment with the two drugs, the CAP and LSM of the patients decreased, which confirmed that both drugs can improve elasticity and fat content of the liver of the patients. To summarize, both metformin and liraglutide can significantly improve the general health of patients with NAFLD, regulate their glucose and lipid metabolism, decrease the levels of inflammatory indicators, and improve liver functions and fat content. The therapeutic effect of liraglutide is better than that of metformin. However, systematic research combining clinical data with the intestinal microbiome is still lacking.

While monitoring the clinical data of patients, we also observed the changes in the intestinal bacterial community. The results showed that both drugs improved the α diversity of the community, and liraglutide significantly increased the diversity and richness of the bacterial community (*p* < 0.05), which were close to those in the HC (Figures 1A,B). The change of intestinal flora diversity is consistent with previous research results (Wang et al., 2017; Tong et al., 2018). The results of β diversity also distinctly showed the efficacy of the two drugs in restoring it to the normal level (Figure 1C). Notably, both drugs showed distinct effects on the intestinal bacterial community of the patients. At the phylum level, the relative abundance of Bacteroidetes in patients treated with metformin increased significantly, whereas liraglutide significantly affected the relative abundance of Actinobacteria. Bacteroidetes can carry leptin, and the increase in its abundance can reduce energy intake, which can affect carbohydrate fermentation and lipopolysaccharide metabolism (Sharpton et al., 2018). Previous studies have shown that the increase in the Firmicutes/Bacteroidetes ratio decreases the production of short-chain fatty acids (SCFA) and increases energy intake, which promotes NAFLD progression (Leung et al., 2016; Yao et al., 2022). The difference is that the abundance of Actinobacteria (mainly *Bifidobacterium*) can increase the production of antibiotics and block the specific binding sites of pathogenic bacteria and toxins (Li et al., 2012). In addition, it can enhance the ability of carbohydrate degradation, increase the production of SCFAs, reduce fat accumulation, and relieve NAFLD-related pathological phenotype (Ni et al., 2020; Oliver et al., 2021). In this study, liraglutide and metformin both increased the relative abundance of Firmicutes, Bacteroidetes, and Actinobacteria, whereas the relative abundance of Proteobacteria containing many pathogenic bacteria decreased. The ratios of Firmicutes/Bacteroidetes decreased from 5.47 (MetA) and 5.29 (LiraA) to 3.53 (MetB) and 3.71 (LiraB), respectively. The increase in SCFA production can improve the lipid metabolism in patients as reported in previous studies (Aron-Wisnewsky et al., 2012; Carmody et al., 2015).

At the genus level, the effect of the two drugs on the structure of the intestinal bacterial community of patients is well-known. Studies have shown that *Escherichia*–*Shigella* species can increase endotoxemia, produce endogenous ethanol, trigger strong inflammatory reactions, and cause insulin resistance (Frost et al., 2021; Zhang et al., 2022). *Megamonas* is closely associated with inflammatory bowel disease, colorectal cancer, and obesity (Chiu et al., 2014; Yachida et al., 2019). In our study, *Escherichia*–*Shigella*, *Megamonas*, and *Bacillus* were considered typical proinflammatory bacteria (Martens et al., 2018; Girinathan et al., 2021), and their relative abundance decreased after the treatment with both drugs, which is consistent with the results of Wang et al. (2017). At the same time, *Faecalibacterium* and *Bifidobacterium*, as traditional probiotics (Yao et al., 2022), showed increased relative abundance after treatment with the two drugs. The difference between the two treatments was that metformin distinctly increased the relative abundance of probiotic *Agathobacter* and liraglutide significantly increased the relative abundance of *Bifidobacterium*, *Dialister*, and *Alistipes*. These results showed that although both drugs can improve the structure of the intestinal flora of the patients, their effects can differ. In addition, LEfSe could more distinctly show that there are obvious differences between the effect of the two drugs at multiple classification levels. The network analysis further showed the effect of the two drugs on the intestinal bacterial network of the patients. Unexpectedly, after metformin treatment, the bacterial network in the intestines of the patients gathered, the proportion of negative correlation edges increased, and the network stability and complexity increased. This indicated that metformin treatment can “compulsorily” unify the bacterial community in the intestines of the patients. Liraglutide has a relatively weaker effect on the intestinal bacterial network of patients, which is similar to that of the healthy group. In terms of therapeutic efficacy, liraglutide is superior to metformin. At the same time, it is milder and more efficient in improving the intestinal community structure of patients.

The present study highlights the clinical data of patients before and after the treatment with the two drugs and determined the changes in the intestinal bacterial community structure. The results showed that the degree of correlations among the β-cell function index, levels of inflammation-related factors, lipid metabolism index, liver function index, and bacterial community (subcommunity) differed significantly after the treatment with the two drugs. For instance, after metformin treatment, HOMA-IR was significantly positively correlated with sub-3, whereas IL-6 was significantly positively correlated with sub-3. However, after liraglutide treatment, HOMA-IR was negatively correlated with sub5, and IL-6 was negatively correlated with sub5. Notably, no significant correlation was observed between HOMA-IR and IL-6 and sub4 (similar to sub5) after metformin treatment. Moreover, for lipid metabolism, the correlation between intestinal bacteria and LDL-C was more significantly positive after metformin treatment, which was contrary to the results of liraglutide treatment. A similar pattern was observed for indicators such as HbA1c, FINS, and ALP. Moreover, based on the matrix correlation analysis, we confirmed the mechanism of metformin and liraglutide treatments on the correlation between intestinal bacterial community and clinical indicators differ significantly, which is consistent with the results of Wang et al. (2017).

Several metabolites of intestinal flora can alter intestinal homeostasis, which can directly or indirectly affect the metabolic processes of bile acids, improve insulin resistance, and produce SCFAs, leading to the occurrence and development of NAFLD (Verhaar et al., 2020; Martin-Gallausiaux et al., 2021). Bile acid and its metabolites help maintain the steady state of TC and TG (Li et al., 2020). Clinical studies have shown that bile acids regulate glucose and lipid metabolism and the production of inflammatory factors, such as IL-6 and TNF-α, in the liver and other tissues through signaling pathways such as farnesol X receptor (Chen et al., 2019; Sinha et al., 2020). In this study, both drugs decreased the abundance of the functional genes of bile acid (Figure 7), which is consistent with the results of previous studies (Smits et al., 2016; Sun et al., 2018; Garzel et al., 2020; Lei et al., 2022). At the same time, our results showed that both drugs increased the abundance of propanoate metabolism functional genes. The difference was that butanoate metabolism increased significantly after liraglutide treatment but decreased after metformin treatment. Studies have shown that the increase in propionic acid contents can promote gluconeogenesis in the liver and intestine, which can be helpful for the treatment of obesity and insulin resistance (Ziętek et al., 2021). Butyric acid plays a crucial role in maintaining intestinal integrity by upregulating the expression of tight junction protein and mucin, which can improve intestinal barrier function and prevent toxic compounds (such as proinflammatory molecules) from migrating to the liver and inhibiting cholesterol synthesis, thereby decreasing liver fat accumulation and regulating the development of NAFLD (Pirola and Sookoian, 2021; Sun et al., 2021). In addition, the treatment with the two drugs decreased the incidence of type 1 diabetes mellitus, T2DM, insulin resistance, glucagon signaling pathway, and nonalcoholic fatty liver disease (NAFLD; Figure 7). This showed the potential role of liraglutide and metformin in the treatment of T2DM complicated with NAFLD based on the intestinal bacterial community.

## Conclusion

Both metformin and liraglutide can be used for the treatment of patients with NAFLD having T2DM, and liraglutide plays a role in decreasing weight, lowering blood sugar level, regulating lipid metabolism, decreasing inflammation, and improving liver function. Both drugs can improve the diversity and richness of the intestinal bacterial community but have distinct effects on the structure of the intestinal bacterial community at multiple classification levels; however, the effect of liraglutide is relatively weak. Metformin or liraglutide treatment exhibits distinct differences in the correlation between intestinal bacterial community and clinical indicators and the effective role of functional gene abundance. However, this study has a limitation of a small sample size, which led to slightly insufficient representation. Studies with a larger sample size are needed to verify and further explore the mechanisms *via* metabonomics.

## Data availability statement

The datasets presented in this study can be found in online repositories. The names of the repository/repositories and accession number(s) can be found at: https://www.ncbi.nlm.nih.gov/, PRJNA896892.

## Ethics statement

The studies involving human participants were reviewed and approved by Ethics Committee of the First Affiliated Hospital of Xinjiang Medical University (Xinjiang Uygur Autonomous Region, China; No. 20181129-13). The patients/participants provided their written informed consent to participate in this study. The animal study was reviewed and approved by Ethics Committee of the First Affiliated Hospital of Xinjiang Medical University (Xinjiang Uygur Autonomous Region, China; No. 20181129-13).

## Author contributions

XY and ZR designed the study and wrote the manuscript. MK, JC, and MW designed and performed the experiments. XY and MK analyzed the data. All authors contributed to the article and approved the submitted version.

## Funding

This study was supported by the Natural Science Foundation of Xinjiang (2017D01C343).

## Conflict of interest

The authors declare that the research was conducted in the absence of any commercial or financial relationships that could be construed as a potential conflict of interest.

## Publisher’s note

All claims expressed in this article are solely those of the authors and do not necessarily represent those of their affiliated organizations, or those of the publisher, the editors and the reviewers. Any product that may be evaluated in this article, or claim that may be made by its manufacturer, is not guaranteed or endorsed by the publisher.
